# Identification of RAPD Markers Linked to Digestive Amylase Genes using Near Isogenic Lines of the Silkworm, *Bombyx mori*

**DOI:** 10.1673/031.010.8401

**Published:** 2010-07-01

**Authors:** S.K. Ashwath, S. Sreekumar, J.T. Toms, S.B. Dandin, C.K. Kamble

**Affiliations:** Genetics and Molecular Biology Laboratory, Central Sericultural Research and Training Institute, Srirampura, Manandavadi Road, MYSORE - 570 008, Karnataka, India

**Keywords:** backcrossing, cathodic amylase

## Abstract

Digestive amylase has been identified as a useful marker for breeding in the silkwrom, *Bombyx mori* L (Lepidoptera: Bombycidae), due to its wide genetic divergence, its role in better digestibility and robustness. The low yielding indigenous *B. mori* breeds of tropics like India are characterized by high activity amylase genes controlled by Amy d^iv^ or d^v^ alleles, while the high yielding breeds of temperate origin are endowed with ‘null’ type (Amy d^n^) with low activity. For improving the digestibility and survival of temperate breeds of Japanese origin, Near Isogenic Lines (NILs) were developed introgressing the Amy d^iv^ and d^v^ alleles from the Donor Parents (DPs) into the genetic background of the Recurrent Parents (RPs) with ‘null’ type of amylase, which showed significant improvement in viability of the NILs. With the objective to know whether the amylase gene itself may confer higher survival by improving digestibility or some other closely linked genes flanking the amylase locus is responsible for better viability of the NILs, RAPD profiles among six *B. mori* breeds comprising of the DPs, RPs, and NILs developed through introgression of Amy d^iv^ or d^v^ alleles were analysed using 27 sets of RAPD primers. Out of the 27 primers, six (OPA01, OPA06, OPA09, OPA15, OPAH03, and OPAH05) showed RAPD products linked to the amylase genes of the DPs introgressed in the NILs, which were absent in their respective RPs. Three amplicons of 1584 bp, 1904 bp, and 1232 bp were specific to Amy d^iv^ allele and one amplified product of 1776 bp was found to be linked with the Amy d^v^ allele. Interestingly, two PCR products of 2628 and 1375 bp were associated with both Amy d^iv^ and d^v^ alleles. The results are discussed in light of further characterization of these amplified products leading to identification of DNA sequences that may be responsible for better digestibility and higher survival in *B. mori.*

## Introduction

The importance of digestive amylase in improving the dietary efficiency in the silkworm, *Bombyx mori* L (Lepidoptera: Bombycidae), was highlighted by Gamo (1983), who showed that the *B. mori* strains with high amylase activity showed higher growth and survival than the low activity strains when the larvae were reared on hardened leaves. Hara et al. (1984, 1986) analysed the activity and amylase isozyme polymorphism in the digestive juice in 361 *B. mori* strains and reported that the digestive amylase gene is located on the eight linkage group (locus 8.3) with three multiple alleles, namely, Amy d^v^ (with five active bands), Amy d^iv^ (with four bands), and Amy d^n^ (null type) due to lack of cathodal isozymes. The *B. mori* strains with Amy d^v^ and d^iv^ alleles had many fold high activity than the Amy d^n^ strains. Studies on Indian *B. mori* strains have also shown that the indigenous polyvoltine strains are carrying the high activity alleles of either Amy d^v^ or d^iv^, while the exotic bivoltine strains are of Amy d^n^ with negligible amylase activity (Patnaik and Datta 1995; Chatterjee et al. 1992). Abraham et al. (1992) analysed the starch content in the fecal matter and reported that low amylase activity strains excreted fivefold more starch than the high activity strains. Correlation studies between four yield attributes and six biochemical parameters among 54 *B. mori* strains has indicated close association of digestive amylase activity with survival, which was not affected by other enzymes (Chatterjee et al. 1993). These studies had clearly shown the prospects of using digestive amylase as a marker in *B. mori* breeding due to its wide genetic diversity, its role in better digestibility, and its higher survival (Datta and Ashwath 2000). To confirm these findings, a breeding scheme was designed and high activity amylase genes from the indigenous, low-yielding polyvoltine donor parents (DP) were introgressed into the productive bivoltine breeds used as recurrent parents (RPs) and Near Isogenic Lines (NIL) of the RPs have been developed (Ashwath et al. 2001). As the evolved NILs have shown higher survival than the RPs, the amylase gene itself may confer higher survival by improving digestibility, or some other closely linked genes flanking the amylase locus may be responsible for better viability of the NILs. Against this background, the RAPD profile among the DPs, RPs, and NILs were analysed with the objective of identification of DNA markers linked to and bordering the amylase locus paving the way for further characterization of chromosome segments conferring higher survival in the evolved lines.

## Materials and Methods

### *B. mori* breeds used

Six *B. mori* breeds comprising of two donor parents (DPs), two recurrent parents (RPs), and the two evolved near isogenic lines (NILs) were used in the present study. The DPs were the indigenous polyvoltine *B. mori* breeds, namely, pure Mysore with Amy d^iv^ allele (four-band type) and Nistari with Amy d^v^ allele (five-band pattern) of cathodic amylase isozyme types. The RPs used were the two productive bivoltine breeds, viz., CSR2 and CSR5, with Amy d^n^ allele (null pattern) of amylase. GEN1 (evolved line of CSR2) with the four-band pattern and GEN2 (evolved line of CSR5) with the five-band pattern, which were derived by introgressing the Amy d^iv^ and d^v^ alleles from the polyvoltine donors i.e., pure Mysore and Nistari, respectively, were the NILs selected for the present investigation.

### Rearing of *B. mori*

The six *B. mori* breeds were reared under 12 crops during 2005–2007, and the data on the survival, yield, cocoon, and post-cocoon parameters were collected. Mean and standard deviations were estimated for the 12 rearings.

### Quantitative and qualitative assay for amylase

Digestive juice samples from the six *B. mori* breeds were collected on the fourth day of the fifth instar by brief exposure of larva to chloroform vapors resulting in instantaneous vomiting. Amylase activity was assayed following the method of Chatterjee et al. (1993) by incubating 10 µl of digestive juice in 0.2% starch as the substrate for 30 min at 37° C, and the reaction was stopped using 1% dinitrosalicylic acid followed by heating in boiling water for 5 min. Amylase assay was carried out in 5 replications for each *B. mori* breed. Optical density values were recorded at 525 nm, and the enzyme activity was expressed as mg of maltose released per ml for 30 min. Mean and standard deviations were estimated for the 5 replications. For qualitative analysis, vertical Polyacrylamide gel electrophoresis was carried out adopting the procedure of Hara et al. (1986) with minor modifications, and cathodic fractions were separated by reversing the polarity of the electrodes. After being run for 6–7 h, the gels were incubated in 1% starch solution for 30 min and subsequently stained with iodine solution for 5 min.

### DNA extraction, PCR amplification and electrophoresis

Genomic DNA from the six *B. mori* breeds, viz., pure Mysore, Nistari, CSR2, CSR5, GEN1 and GEN2, was extracted from the whole larval body of fifth instar larvae using the phenol-chloroform method and purified using the standard procedure of Sambrook and Russel (2001). The purified DNA was diluted to a final concentration of 20 ng/µl. PCR reactions were carried in a PTC 200 gradient thermal cycler (MJ Research, www.mjr.com) using the 27 sets of RAPD primers for amplifying the DNA of the *B. mori* breeds (Table 3). Amplifications were carried out in 25 µl reactions containing 1x reaction buffer (100 m*M* Tris, 500 m*M* KCl, 0.1% gelatin, pH 9.0), 1.5 *mM* MgCl_2_, 6–8 picomoles of primer, 200 µ*M* dNTPs, 2 U Taq DNA polymerase, and 20 ng of template DNA. A negative control was set up omitting the DNA from the reaction mixture. The reaction mixture was pre-heated at 95° C for three min, followed by 40 cycles (94° C for 3 min, 40° C for 1 ½ min, and 72° C for 2 min). A final extension for 10 min at 72° C was given after the completion of the cycles. Resulting PCR products were electrophoretically analyzed through 1.5% agarose gels containing ethidium bromide (5 µg/ml) in 1x TAE buffer (pH 8.0) and documented in the gel documentation system (Syngene, www.syngene.com). A molecular weight marker (lambda with EcoR1 / Hind III double digest) was used for the analyses of the fragment size. All amplification reactions were carried out at least thrice in order to ensure consistency and repeatability of fingerprints generated using selected RAPD primers.

## Results and Discussion

Near isogenic lines (NIL) have been evolved in many crop plants, including tomato, rice, wheat, barley, soybean, etc., for introgressing disease resistance genes from the wild relatives into the cultivated varieties (Young and Tanksley 1989). NILs have been developed through recurrent backcross breeding procedure where a hybrid (F1) is raised between the donor parent (DP) and the recurrent parent (RP) and subsequently the RP is repeatedly backcrossed. At each generation, the progeny is selected for the target gene of the DP prior to each backcrossing. During each backcross, the genetic background of the RP is increased by 50% and the genome of the selected individuals progressively becomes almost exclusively of that of the RP with a small segment of DNA of the DP flanking the target gene. Thus the products of introgression results in a pair of NILs that are identical except for a region near the target gene. Most of the NILs developed so far have used five to six backcrosses.

The indigenous polyvoltine *B. mori* breeds used as DPs, namely, pure Mysore and Nistari, have been reared in India for more than 100 years, the former being popular in south India and the latter in north India. The survival potential of these breeds, which are being reared throughout the year, are high, but these polyvoltines are characterized by lower cocoon wt., shell wt, with lower silk content, shorter filament length, and poor silk quality. The RPs (CSR2 and CSR5) are the evolved *B. mori* breeds extracted from the Japanese double hybrids, fixed and acclimatized to the Indian tropical condition (Datta et al. 2000). Subsequently, these breeds were authorized for commercial use, which are highly productive with higher cocoon wt, shell wt. with high raw silk recovery, longer filament length and better silk quality. However, in view of superior quantitative traits, rearing of these productive breeds needs more care and attention, as they are susceptible under the high germ load and unfavorable climatic conditions of tropics leading to low pupation rate and crop failures. Hence, rearing of seed cocoons is possible only in selected seasons with elite farmers who can provide the required inputs and optimum rearing conditions.

For improving their survival potential of the productive bivoltine *B. mori* breeds, the new strategy of molecular marker assisted selection in breeding was employed using digestive amylase as a marker. During the breeding process, productive *B. mori* breeds, CSR2 and CSR5 with “null” type (Amy d^n^ allele) with low amylase activity were selected as Recurrent Parents (RPs) and the indigenous polyvoltine breeds, Pure Mysore (with four-band Amy d^iv^ allele) and Nistari (with five-band Amy d^v^ allele), both having high amylase activity, were selected as Donor Parents (DPs). F1s were raised between the DPs and RPs, and the F1 progeny were backcrossed to their respective RPs. In each generation, the BC progeny were screened by amylase assay. Only those with the DP type of amylase were selected for further backcrossing, and the progeny with “null” type were rejected. This selection procedure was repeated for ten generations of backcrossing with the RPs, to incorporate the higher productivity traits of RPs, simultaneously selecting the high activity amylase genes of the DPs. The BC10 progeny were selfed twice followed by test crossing with the “null” type to derive the homozygous NILs. This breeding scheme has resulted in the evolution of two NILs, namely GEN1 and GEN2, which have been introgressed with high activity amylase genes (Amy d^iv^ and Amy d^v^ alleles, respectively), simultaneously retaining the productivity traits on par with those of their respective RPs. As ten backcrosses have been carried out, theoretically in the NILs, the RP recovery is assumed to be about 99.95%, retaining all the productivity traits, while the DP genome is reduced to 0.05%, eliminating all the undesirable traits except the high activity amylase genes. The detailed breeding procedure has been reported previously (Ashwath et al. 2001).

The donor parents (DPs) and the recurrent parents (RPs) used in the present investigation are showing large differences for the economic traits (Table 1). The values indicated under yield/10,000 larvae by number is a measure of survival, which denotes the number of pupated cocoons harvested out of the 10,000 larvae retained after III moult. From the table, it is evident that the indigenous DPs have shown higher survival than the RPs, whereas the productivity traits like cocoon weight, shell weight, shell (%), filament length and raw silk (%) of the RPs are almost two fold higher than that of the DPs. Both the NILs have shown more than 10% higher survival than that of their respective RPs, while the productivity traits are on par with those of the RPs, in spite of using low yielding polyvoltines as DPs.

**Figure 1.**
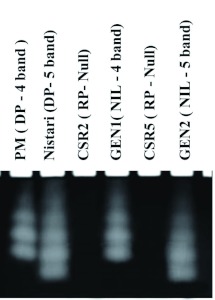
Cathodic digestive amylase isozyme pattern in the Donor Parents (DPs), Recurrent Parents (RPs) and Near Isogenic Lines (NILs). DPs, Pure Mysore (PM) has ‘4 band’ type, Nistari has ‘5 band type’, the RPs, CSR2 and CSR5 have ‘null’ type and the NIL of CSR2 - GEN1 shows ‘4 band’ pattern and the NIL of CSR5 -GEN2 has ‘5 band’ phenotype. High quality figures are available online.

The quantitative assay for amylase (Table 2) has shown more than two fold higher activity in the DPs (172.9 to 206 mg/ml) than that of the RPs (80 to 83 mg/ml). Further, the NILs have almost regained the activity of the DPs in spite of backcrossing ten times with the RPs with low amylase activity. The qualitative assay by cathodic PAGE (Figure 1) has revealed either the four-band (pure Mysore) or five-band (Nistari) isozyme pattern in the DPs, while in the RPs (both CSR2 and CSR5) it is of null type. The NILs (GEN1 and GEN2) clearly show the isozyme profiles derived from their respective DPs, namely, pure Mysore and Nistari.

**Table 1.**
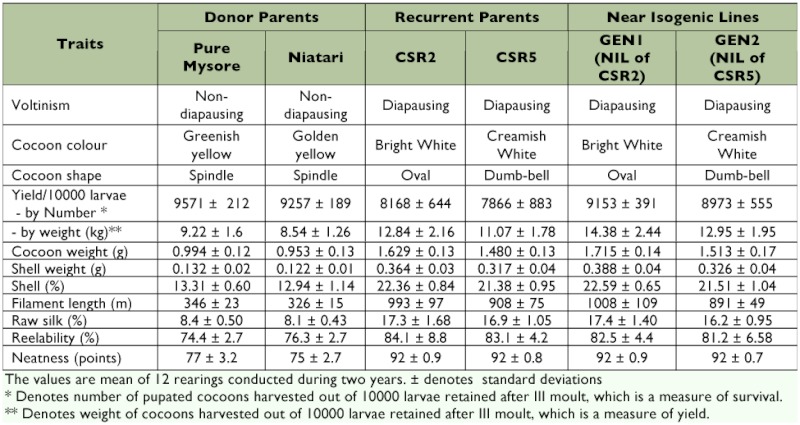
Characteristics of the silkworm breeds used.

Use of NILs have been found to be effective in identifying, from a large number of DNA markers, the few that reside in the vicinity of the target gene in relatively short time. The near isogenecity of the NTL to its RP provides a unique resource to identify whether a genomic clone is located near the gene of interest. The strategy of identification of DNA markers linked to the target gene involves screening of the DNA markers between the DP and RP and identification of polymorphic markers. These informative primers will be analysed using the DP/NIL/RP trio sets. The sets showing RP/NIL allelic contrast and corresponding NIL/DP allelic equality for the informative DNA marker will establish the linkage of the marker with the target trait. NILs have been extensively used in crop plants for rapidly identifying the DNA markers that are tightly linked to the gene of interest, like tobacco mosaic virus resistance gene (Young and Tanksley 1989), *Pseudomonas* resistance gene (Martin et al. 1991) in tomato, stripe resistance genes (Coram et al. 2008; Yan et al. 2003; Chague et al. 1999), leaf rust resistance genes (Gupta et al. 2005; Thatcher et al. 2003), powdery mildew resistance genes (Ma et al. 2004) in wheat, QTLs for root traits (Steele et al. 2006), chlorophyll content (Wang et al. 2008), bacterial blight resistance genes (Gu et al. 2008) in rice and barley (Nduulu et al. 2007), and maturity genes in soybean (Matsumara et al. 2008). Similarly, in the present investigation, NILs developed in *B. mori* has been used for detecting DNA markers associated with high activity digestive amylase genes.

**Table 2.**
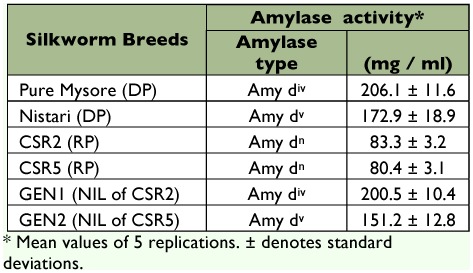
Profile of digestive amylase in the parents and NILs

The template DNA from the DPs, RPs, and NILs were amplified with 27 sets of RAPD primers (Table 3) and the amplicons were viewed in 1.5% ethidium bromide stained agarose gels. A total of 178 amplified products were scored, out of which 43 were monomorphic, and the remaining 135 were polymorphic. The number of bands scored for each primer varied from three (OPAA2, OPAH2) to as high as 12 (OPA4, OPA8), and the average number of bands per primer was 6.59. Very high degree of polymorphism ranging from 100% (OPA6, OPA7, OPA17, OPAA2, and OPAH3) to 42.8% (OPA5) was observed. The overall polymorphism with 27 primers was found to be 75.8%.

**Figure 2.**
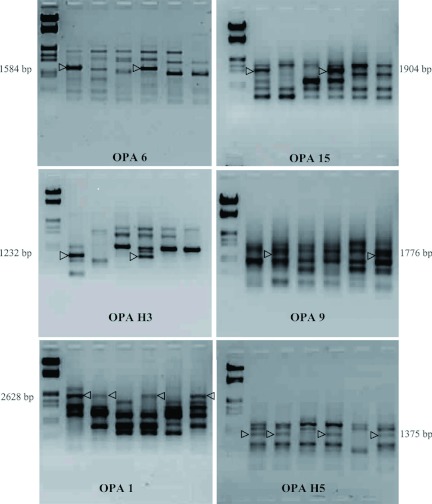
DNA profiles of DPs, RPs and NILs amplified with 6 RAPD primers showing RAPD products linked to the amylase gene of the DPs introgressed in the NILs. Note their absence in the respective RPs. High quality figures are available online.

Out of the 27 primers used, six primers (OPA1, OPA6, OPA9, OPA15, OPAH3, and OPAH5) showed RAPD products linked to the amylase genes of the DPs introgressed in the NILs, which were absent in their respective RPs (Figure 2 and Table 4). It may be mentioned here that the DPs, namely, pure Mysore (PM), has the four-band type of isozyme, and Nistari has the five-band pattern, which are the alleles (Amy d^iv^ and Amy d^v^, respectively) of the same digestive amylase gene Amy d located at 2.8 cM on the 8^th^ linkage group (Doira 1992). When OPA6, OPA15, and OPAH3 were used for amplifying the DNA of the six breeds, amplicons of 1584 bp, 1904 bp, and 1232 bp were detected in the PM (DP) and its NIL GEN1, which were totally absent in the RP, CSR2 and also in the DP/ RP/ NIL trio set of Nistari/CSR5/GEN2. This clearly indicates the close linkage of these RAPD products with Amy d^iv^ allele. Similarly, OPA9 primer detected the PCR product of 1776 bp present only in Nistari (DP) and its NIL, GEN2, which was not found in its RP, CSR5 as well as the DP/RP/NIL trio set of PM, CSR2, and GEN1. This strongly supports the possibility of a close linkage of this PCR product with the Amy d^v^ allele. Interestingly, in the case of OPA1 and OPA H5, PCR product of 2628 and 1375 bp respectively were detected both in the DPs, PM, and Nistari as well as their respective NILs, GEN1, and GEN2, while these bands were absent in their respective RPs, CSR2, and CSR5. This indicates that these PCR amplicons are associated with both Amy d^iv^ and d^v^ alleles. At this stage, it is not possible to substantiate how some PCR products are linked to Amy d^iv^ and Amy d^v^ separately, and few are specific to both Amy d^iv^ and d^v^ alleles together. Further studies involving cloning, sequencing, and detailed characterization of these amplified products may be required to understand whether these are parts of the amylase gene itself or closely linked sequences flanking the amylase gene. As the NTLs evolved by introgressing the digestive amylase genes have shown better viability than their respective RPs, characterization of the PCR products closely associated with the amylase gene can lead to identification of DNA sequences that may be responsible for better digestibility and higher survival in *B. mori* which could be used for the development of improved and robust *B. mori* breeds for commercial exploitation through the strategy of DNA marker assisted selection.

**Table 3.**
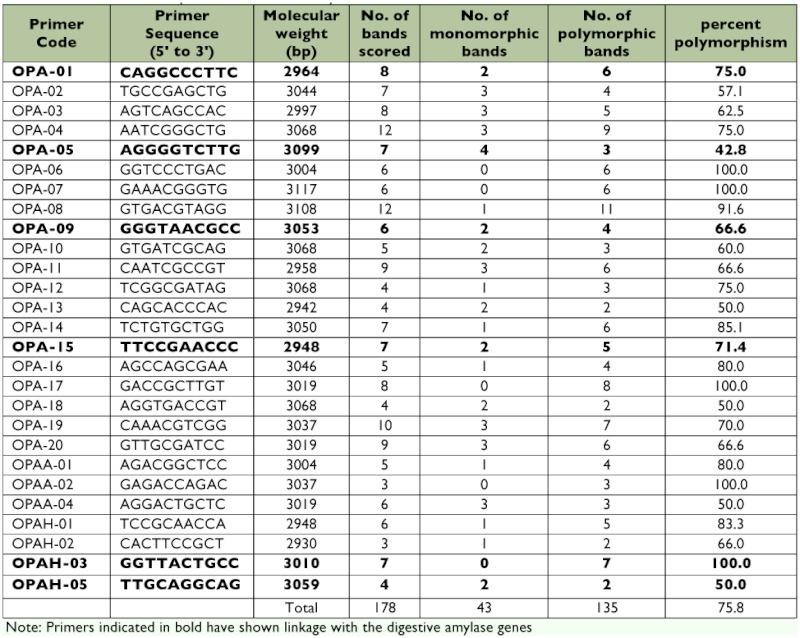
Details of RAPD primers used in the analysis.

**Table 4.**
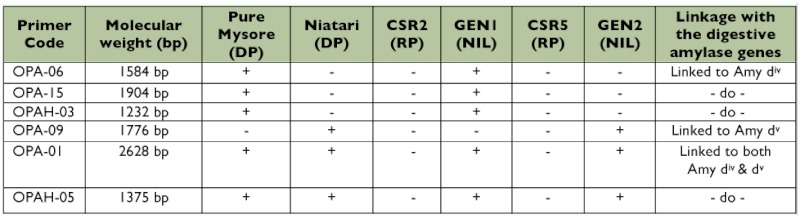
RAPD products linked to amylase genes of the DPs introgressed in the NILs

## Abbreviatons

DP: donor parents;
NIL: near isogenic lines;
PM: Pure Mysore;
RP: recurrent parents

## References

[bibr01] Abraham EG, Nagaraju J, Datta RK (1992). Biochemical studies of amylases in the silkworm, *Bombyx mori* L.: Comparative analysis in diapausing and nondiapausing strains.. *Insect Biochemistry and Molecular Biology*.

[bibr02] Ashwath SK, Morrison MN, Datta RK (2001). Development of near isogenic lines of productive silkworm breeds by isozyme marker based selection.. *Proceedings of National Academy of Sciences, India*.

[bibr03] Chague V, Fahima T, Dahan A, Sun GL, Korol AB, Ronin YI, Grama A, Roder MS, Nevo E (1999). Isolation of microsatellite and RAPD markers flanking the Yr15 gene of wheat using NILs and bulked segregant analysis.. *Genome*.

[bibr04] Chatterjee SN, Rao CGP, Chatterjee GK, Ashwath SK (1992). Genetic variability of amylase activity in the mulberry silkworm, *Bombyx mori* L. and its significance.. *Sericologia*.

[bibr05] Chatterjee SN, Rao CGP, Chatterjee GK, Ashwath SK, Patnaik AK (1993). Correlation between yield and biochemical parameters in the mulberry silkworm, *Bombyx mori* L.. *Theoretical and Applied Genetics*.

[bibr06] Coram TE, Settles ML, Wang M, Chen X (2008). Surveying expression level polymorphism and single-feature polymorphism in near-isogenic wheat lines differing for the Yr5 stripe resistance locus.. *Theoretical and Applied Genetics*.

[bibr07] Datta RK, Ashwath SK (2000). Strategies in genetics and molecular biology for strengthening silkworm breeding.. *Indian Journal of Sericulture*.

[bibr08] Datta RK, Basavaraja HK, Mal Reddy N, Nirmal Kumar S, Ahsan MM, Suresh Kumar N, Ramesh Babu M (2000). Evolution of new productive bivoltine hybrids CSR2 × CSR4 and CSR2 × CSR5.. *Sericologia*.

[bibr09] Doira H (1992). *Genetical stocks and mutations of Bombyx mori: Important genetic resources.*.

[bibr10] Gamo T (1983). Biochemical genetics and its applications to the breeding of the silkworm.. *Japanese Agriculture Research Quarterly*.

[bibr11] Gu K, Sangha JS, Li Y, Yin Z (2008). High resolution genetic mapping of bacterial blight resistance gene Xa 10.. *Theoretical and Applied Genetics*.

[bibr12] Gupta SK, Charpe A, Koul S, Prabhu KV, Haq QM (2005). Development and validation of molecular markers linked to an *Aegilops umbellulata*-derived leaf rust resistance gene, Lr9 for marker assisted selection in bread wheat.. *Genome*.

[bibr13] Hara W, Fujii H, Sakaguchi B (1986). The *ae* locus and the genes controlling the cathodal amylase isozymes of digestive juice in *Bombyx mori*.. *Journal of Sericulture Science of Japan*.

[bibr14] Hara W, Fujii H, Sakaguchi B (1984). Activity and polymorphism of digestive juice amylase in various strains of *Bombyx mori*.. *Journal of Sericulture Science of Japan*.

[bibr15] Ma ZQ, Wei JB, Cheng SH (2004). PCR based markers for the powdery mildew resistance gene Pm4a in wheat.. *Theoretical and Applied Genetics*.

[bibr16] Martin GB, Williams JG, Tanksley SD (1991). Rapid identification of markers linked to *Pseudo-monas* resistance gene in tomato by using random primers and near isogenic lines.. *Proceedings of National Academy of Sciences USA*.

[bibr17] Matsumara H, Liu B, Abe J, Takahashi R (2008). AFLP mapping of soybean maturity gene E4.. *Journal of Heredity*.

[bibr18] Nduulu LM, Mesfin A, Muehlbauer GJ, Smith KP (2007). Analysis of chromosome 2(2H) region of barley associated with the correlated traits, *Fusarium* head blight resistance and heading date.. *Theoretical and Applied Genetics*.

[bibr19] Patnaik AK, Datta RK (1995). Amylase-its genetics and prospects as a marker in silkworm breeding.. *Indian Journal of Sericulture*.

[bibr20] Sambrook J, Russel DW (2001). *Molecular Cloning, A Laboratory Manual*.

[bibr21] Steele KA, Price AH, Shashidhar HE, Witcombe JR (2006). Marker assisted selection to introgress rice QTLs controlling root traits into an Indian upland rice variety.. *Theoretical and Applied Genetics*.

[bibr22] Thatcher CJ, Goika L, Stepien L (2003). Application of STS markers for leaf rust resistance genes in near isogenic lines of spring wheat.. *Journal of Applied Genetics*.

[bibr23] Wang F, Wang G, Li X, Huang J (2008). Heredity, physiology and mapping of a chlorophyll content gene of rice (*Oryza sativa* L).. *Journal of Plant Physiology*.

[bibr24] Yan GP, Chen XM, Line RF, Wellings CR (2003). Resistance gene-analog polymorphism markers co-segregating with the YR5 gene for resistance to wheat stripe rust.. *Theoretical and Applied Genetics*.

[bibr25] Young ND, Tanksley SD (1989). RFLP analysis of the size of chromosomal segments retained around the *Tm-2* locus of tomato during backcross breeding.. *Theoretical and Applied Genetics*.

